# Nutcracker Syndrome with Hypertension: A Case Report

**DOI:** 10.7759/cureus.4781

**Published:** 2019-05-30

**Authors:** Azhara Binte Azhar, Nain Tara Zeb, Sabeeka Shah, Amnah Khalid

**Affiliations:** 1 Internal Medicine, Shifa College of Medicine, Shifa International Hospital, Islamabad, PAK; 2 Public Health, Shifa College of Medicine, Shifa International Hospital, Islamabad, PAK

**Keywords:** nutcracker syndrome, hypertension, renal artery stenosis, nutcracker phenomenon

## Abstract

We describe the case of a young female who presented with hypertension, left flank pain, and hematuria. Workup was done to determine secondary causes of hypertension, which indicated renal artery stenosis. However, the renal angiography did not reveal any focal areas of stenosis. Further investigations revealed delayed renal vein emptying, suggesting extrinsic renal vein compression and leading to a diagnosis of nutcracker syndrome.

## Introduction

The nutcracker phenomenon (NCP) refers to the compression of the left renal vein (LRV), most commonly between the aorta and the superior mesenteric artery, with impaired blood outflow often accompanied by distention of the distal portion of the renal vein [1]. NCP can be due to a variety of causes, including renal or ureteric calculi or tumors, intrinsic kidney disease, and loin pain hematuria syndrome [1]. The term nutcracker syndrome (NCS) is used for patients with clinical symptoms while the nutcracker phenomenon describes the anatomical variation without symptoms. Symptoms include hematuria, orthostatic proteinuria, flank pain, abdominal pain, varicocele, dyspareunia, dysmenorrhea, fatigue, and orthostatic intolerance [2]. Typically, hypertension has not been included in the traditional clinical manifestations of nutcracker syndrome. The exact prevalence of NCP is unknown but may be slightly higher in females [3].

## Case presentation

We present the case of an 18-year-old, South Asian female with no known comorbidities, who presented to the outpatient department with a history of hypertension for the past nine months. She had associated inconsistent left flank pain, fatigue, palpitations, and increased urinary frequency, at least twice during the day and four times during the night. Although the patient had these complaints for 12-15 months, they only came into notice on a routine checkup nine months ago. There was no history of recurrent urinary tract infections (UTIs) and no family history of kidney diseases. The patients' ambulatory blood pressure readings revealed continuously elevated blood pressure as high as 200/110 mm Hg.

On examination, her heart rate was 96 bpm, blood pressure was 170/110 mm Hg, and she was afebrile. Abdominal examination was unremarkable except for mild left lumbar tenderness on palpation. There were no other findings.

An extensive workup was done and the secondary causes of hypertension, such as pheochromocytoma, congenital adrenal hyperplasia, renin-secreting tumor, Cushing’s disease, and hyperthyroidism, were ruled out. Urine routine examination revealed albumin 1+, blood 4+, and red blood cells (RBCs) >100/HPF. Doppler ultrasonography (USG) of the kidneys revealed left-sided renal artery stenosis, with the left kidney smaller in size (8 x 3.5 x.1.2 cm) as compared to the right kidney (11 x 3.0 x 1.3 cm). Magnetic renal angiogram (MRA) was performed to correlate the previous findings. It showed a small left kidney (7 x 3 cm) with cortical scarring and diffuse narrowing of the left main renal artery with a small portion of focal stenosis at the level of approximately 9 mm from the ostium.

**Figure 1:**
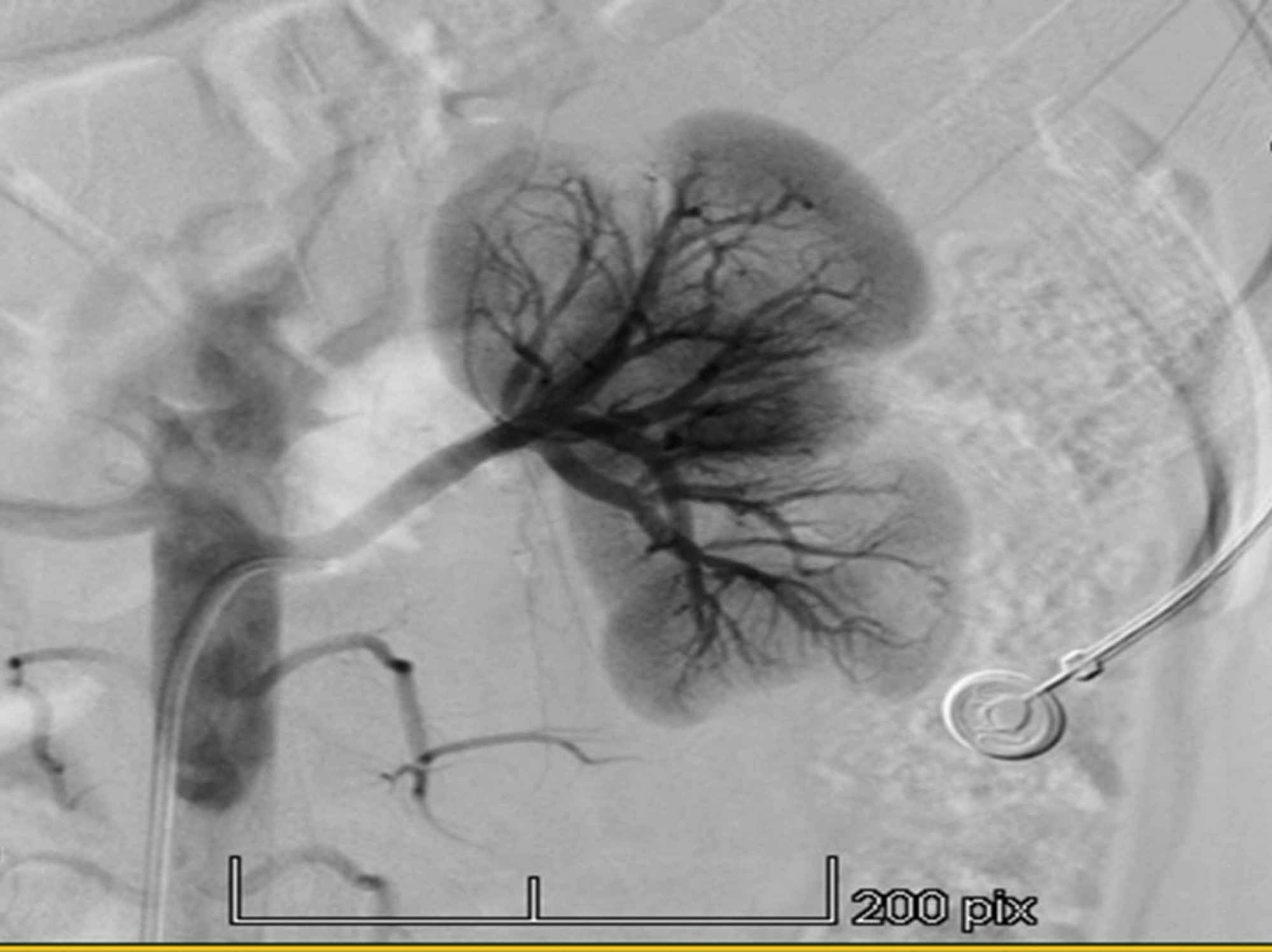
Renal angiogram showing no areas of stenosis in the left renal artery

**Figure 2:**
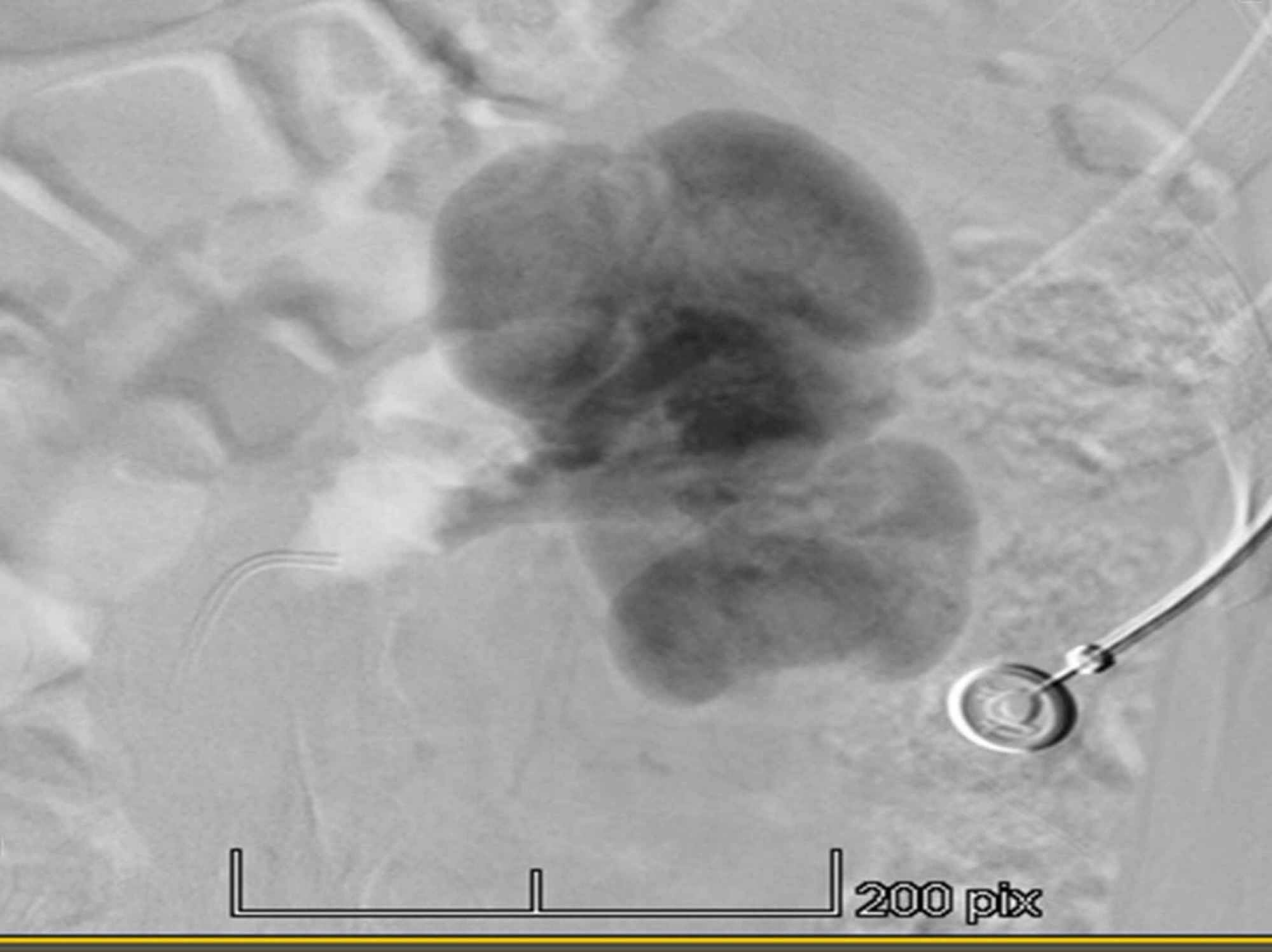
Renal angiogram showing delayed left renal vein emptying

The workup suggested left renal artery stenosis. The patient was admitted to undergo renal angiography and stenting if needed. The renal angiography showed a normal right renal angiogram without any areas of focal stenosis or narrowing and the right renal veins were draining normally. The left renal angiogram showed cortical scarring and a normal left renal artery without any areas of focal stenosis (Figure 1). Delayed left renal vein emptying was seen (Figure 2). These findings were concerning for extrinsic left renal vein compression secondary to the superior mesenteric artery, also known as nutcracker syndrome. The patient was offered a trial of medical management with the possibility of surgical intervention later on. The patient was discharged on valsartan 160 mg twice daily, amlodipine 5 mg once daily, and nebivolol 5 mg once daily to control her hypertension. At her one-month follow-up, her blood pressure was 120/85 mm Hg and heart rate was 73 beats per minute.

## Discussion

Nutcracker syndrome (NCS) occurs because of renal vein compression. There are many different anatomical variants that can lead to renal vein compression, with the most common being compression by the aorta or superior mesenteric artery. NCS can also be classified clinically into two types: typical, with renal manifestations, and atypical, with urologic manifestations. Typical symptoms of NCS include gross hematuria and orthostatic proteinuria, with or without flank pain. Atypical NCS presents with symptoms of fatigue, flank/abdominal pain, orthostatic intolerance, dysmenorrhea and dyspareunia in women, and varicocele in men [4]. Typically, hypertension has not been included in the traditional clinical manifestations of nutcracker syndrome but, as shown in our case, it can be a presenting symptom of NCS.

In this case report, we have presented a case of an 18-year-old female who had persistent hypertension for nine months along with urinary frequency and flank pain. Renal artery stenosis was suspected but subsequent renal artery angiography failed to show any significant stenosis as a cause of her hypertension. Delayed renal vein emptying was observed, and it was concluded that the patient had extrinsic renal vein compression consistent with atypical NCS. The patient was managed with valsartan, nebivolol, and amlodipine with adequate blood pressure control at the one-month follow-up.

The diagnosis of NCS is typically based on a stepwise workup with history and clinical examination, followed by Doppler USG, computed tomography, magnetic resonance imaging, intravascular ultrasound (IVUS), and phlebography, with measurement of the renocaval pressure gradient [5]. A real-time Doppler USG is recommended as the first diagnostic test in patients with suspected NCS. Although Doppler ultrasonography and MRA suggested renal artery stenosis in our patient, renal angiography suggested otherwise.

The management of NCS is determined by symptom severity; often, symptom resolution occurs following a conservative approach. Patients under 18 years of age are usually managed conservatively. Patients with mild hematuria or the spontaneous resolution of hematuria can also be managed conservatively. However, in some cases, such as patients with severe pain and persistent hematuria, surgical management may be required [5]. Open surgery, laparoscopic surgery, stent implantation, and endovascular surgery (EVS) all yield satisfactory results in management [6].

## Conclusions

NCS occurs due to renal vein compression and can present with a range of symptoms. Although there is no clear diagnostic criteria, hypertension is considered an unusual presenting symptom for NCS. This report outlined the case and management of a patient who presented mainly with hypertension due to NCS. The diagnosis of NCS is based on radiographic findings and, in this case, we observed delayed renal vein emptying, indicating external vein compression. The treatment of NCS can be either medical or surgical, depending on patient age and the severity of symptoms. Our patient is being managed medically for hypertension with relief from symptoms. Due to its rarity and lack of clear diagnostic criteria, it is important to investigate NCS in young patients who present with hypertension, to allow reliable diagnosis and management.
